# Cytokines and Chemokines: Disease Models, Mechanisms, and Therapies

**DOI:** 10.1155/2014/296356

**Published:** 2014-07-24

**Authors:** Salahuddin Ahmed, Charles J. Malemud, Alisa E. Koch, Mohammad Athar, Dennis D. Taub

**Affiliations:** ^1^Department of Pharmaceutical Sciences, Washington State University College of Pharmacy, 412 E. Spokane Falls Boulevard, Spokane, WA 99204, USA; ^2^Department of Medicine/Rheumatology, Case Western Reserve University, Cleveland, OH 44106, USA; ^3^Veteran's Administration, Ann Arbor, MI and Department of Internal Medicine/Rheumatology, University of Michigan, Ann Arbor, MI 48109, USA; ^4^Department of Dermatology, University of Alabama at Birmingham, Birmingham, AL 35294, USA; ^5^Department of Veterans Affairs, VA Medical Center, Washington, DC 20422, USA

The mechanistic insights gained through research into cytokine and chemokine pathways have progressed to the point where it has made possible the successful targeting of these factors in order to limit their role in orchestrating a variety of rheumatic, skin, vascular, and infectious diseases. While this has set the stage for a new generation of biological therapies, significant gaps still remain in our understanding of how these cytokines and chemokines actually work at the cellular and molecular level. This gap in knowledge demands that research continue to explore for novel insights into the existing mechanisms of established inflammatory mediators and for the discovery of the possible role of silent or new participants in the processes of acute or chronic inflammation. This special issue invited contributors to submit original research articles or reviews in order to further strengthen the efforts to understand the cellular and molecular mechanisms of cytokine- and chemokine-mediated inflammation in disease pathogenesis. We were particularly interested in papers that provide novel understanding of the role of cytokines, chemokines, or their receptors in disease pathogenesis and testing of therapeutic strategies in cell/tissue culture systems, in animal models, or in the clinical evaluation of inflammatory diseases. We received overwhelming response to the call for papers that went beyond our expectation in terms of not only submission numbers, but also the variety of research that were accepted in this special issue. We would like to thank all the authors for their outstanding contributions and responsiveness to the reviewer's concerns. Our editorial board would also like to thank and warmly acknowledge the reviewers for their service in reviewing these interesting papers.

This special issue is comprised of 26 papers that cover the spectrum of novel findings in this area of research ranging from the basic molecular biology of cytokines and chemokines to some related clinical findings and therapeutic applications. More interesting to the readers will be the breadth of diseases covered in terms of these inflammatory mediators including those relevant to rheumatic diseases, atherosclerosis, multiple sclerosis, pulmonary fibrosis, diabetic retinopathy, kidney diseases, carcinogenesis, inflammatory bowel disease, periodontitis, stroke, and aging.

In the paper entitled “*The pathology of orthopedic implant failure is mediated by innate immune system cytokines*,” S. Landgraeber et al. provide a comprehensive review and update on clinical and basic science studies that are needed to address progressive pathological bone loss or “aseptic loosening” of orthopedic implants and the implications this has as a potentially life-threatening condition especially in the elderly. Based on their summary, the authors presented their viewpoint which discussed the underlying mechanisms for how failed orthopedic implants might contribute to activation of the innate and adaptive immune responses. They also highlighted the role that different components of innate immunity such as cytokines, toll-like receptors, apoptosis, bone catabolism, and hypoxic responses play in the failure of orthopedic implants.

In the paper entitled “*Effect of tumor necrosis factor family member LIGHT (TNFSF14) on the activation of basophils and eosinophils interacting with bronchial epithelial cells*,” H. N. Qiu et al. investigated the underlying mechanisms of the activity of LIGHT in cocultures of human basophils/eosinophils and BAES-2B epithelial cells. Using well-validated techniques, the authors found that LIGHT could significantly promote intercellular adhesion and extracellular matrix (ECM) remodeling in basophil/eosinophil/BAES-2B cell cocultures through the enhanced expression of intercellular adhesion molecule-1 (ICAM-1) as well as via the enhanced synthesis of interleukin-6 (IL-6), CXCL8, and matrix metalloproteinase-9 (MMP-9). An evaluation of the signal transduction mechanisms suggested that LIGHT mediates these negative effects by activating ERK, p38-MAPK, and NF-*κ*B pathways. Overall, these findings provided a firmer understanding of the immunopathological role of LIGHT relevant to allergic asthma and proposed LIGHT as a potential therapeutic target for this disease.

The paper by J. S. Fernandes et al. addressed the mechanism and role of monocyte subsets in the development of periportal fibrosis induced by egg antigens that leads to* Schistosoma mansoni *infection. Using extensive flow cytometric analysis, the authors discovered that the level of the classical subset of monocytes (i.e., CD14^++^CD16^−^) was elevated in the* S. mansoni* patient population and in addition primarily showed higher expression of HLA-DR, IL-6, tumor necrosis factor- (TNF-) *α* and transforming growth factor- (TGF-) *β*, which correlated with moderate to severe fibrosis as compared to the other patient groups. This study differentiated the presence and abundance of different monocyte subsets in the individuals with varying degrees of periportal fibrosis secondary to schistosomiasis. In addition, this analysis also provided an impetus to target the CD14^++^CD16^−^ monocyte subset which should further provide an understanding of their role in the pathogenesis of schistosomiasis and potentially target these cells in the treatment of periportal fibrosis associated with this parasitic infection.

In the research article entitled “*The proinflammatory cytokine high-mobility group box-1 mediates retinal neuropathy induced by diabetes*,” A. M. Abu El-Asrar et al. tested the hypothesis that the increased expression of the proinflammatory cytokine, high-mobility group box 1 (HMGB1), in epiretinal membranes and vitreous fluid from patients with diabetic retinopathy plays an important pathogenic role in rats with diabetes-induced retinal neuropathy. Their data showed that HMGB1 was upregulated early in the development of diabetes in these rodents, which also correlated with the enhanced activation of ERK1/2, active caspase-3 and glutamate levels. Furthermore, their analysis also found a marked downregulation of synaptophysin, tyrosine hydroxylase, glutamine synthetase, and glyoxalase 1. The treatment of these diabetic rats with glycyrrhizin significantly attenuated diabetes-induced HMGB1 upregulation without affecting their metabolic status. These findings suggested that HMGB1 could be an important mediator in the early events that lead to diabetic retinal neuropathy. Thus, HMGB1 could be a potential therapeutic target for the amelioration of diabetic neuropathy.

In the study entitled “*Interactions between neutrophils, Th17 cells, and chemokines during the initiation of experimental model of multiple sclerosis*,” D. W. Wojkowska et al. analyzed the expression of CC and CXC chemokine receptors, Th17 cell activation, and neutrophil migration to the brain in an experimental model of multiple sclerosis (MS). MS is a demyelinating disease affecting the central nervous system (CNS) in which activated T cells and their interaction with neutrophils lead to chronic pathological CNS deterioration. However, the immunopathogenesis of MS and the progression of CNS damage in a validated experimental model of MS, namely, experimental autoimmune encephalomyelitis (EAE), remain unclear despite many decades of active research. Thus, the authors provide evidence for the role of Th17 in upregulating CCR6, CXCR2, and CXCR6 expression which allowed neutrophil accumulation and neuronal damage. This study also provided evidence that anti-IL-23R and anti-CXCR2 antibodies were effective in amelioration of EAE by decreasing the intracerebral accumulation of Th17 cells.

In the paper entitled “*Regulation of chemokine CCL5 synthesis in human peritoneal fibroblasts: a key role of IFN*-*γ*,” E. Kawka et al. studied the potential of IFN-*γ* to synergistically exacerbate IL-1*β* or TNF-*α* responsiveness of stimulated human peritoneal fibroblasts (HPFB) to produce CCL5, a potent chemokine for mononuclear leukocytes. They further showed that interferon-*γ* (IFN-*γ*) induced the expression of CD40 receptor in HPFB cells which led to an enhanced response to CD40L and consequently CCL5 synthesis. The results of this study suggested that HPFB synthesize CCL5 in response to inflammatory mediators, which may contribute significantly to the recruitment of mononuclear leukocytes in peritonitis.

In the article entitled “*Chemokines and chemokine receptors in multiple sclerosis*” by W. Cheng and G. Chen. The authors compiled results from recent studies that provided novel evidence for the role of chemokines and chemokine receptors in multiple sclerosis (MS) pathogenesis. These authors systematically detailed the 4 distinct patterns of human MS, relapsing-remitting, primary progressive, secondary progressive, and primary-relapsing. In light of current results, the authors also discussed how migration of T cells and macrophages from peripheral blood to the CNS and the destruction of blood brain barrier were two of the key pathogenic events where the chemokine/chemokine receptor duality may have pathologic relevance. Based on this contention they suggested that the chemokine network may be a potential therapeutic target for intervention in human MS.

In the review article entitled “*The inflammatory chemokine CCL5 and cancer progression*,” D. Aldinucci and A. Colombatti discussed the importance of chemokines as the “gate keepers” of immunity and inflammation. However, the activity of chemokines is flipped in the tumor microenvironment wherein cancer cells start subverting chemokine networks to their advantage in such a way that these chemokines now exert tumor-promoting effects relevant to carcinogenesis. The authors focused their review on CCL5/CCR5 duo and highlighted newer therapeutic strategies which are aimed at inhibiting the binding of CCL5 to CCR5, thus inhibiting CCL5 secretion or alternatively using this strategy to inhibit interactions between tumor cells and their microenvironment leading to decreased CCL5 secretion.

In the research paper entitled “*CD38 ligation in peripheral blood mononuclear cells of myeloma patients induces release of protumorigenic IL-6 and impaired secretion of IFNγ cytokines and proliferation*,” G. Fedele et al. studied the role of CD38 in impairing T cell immune responses in multiple myeloma (MM) peripheral blood mononuclear cells (PBMCs). The authors employed a monoclonal antibody ligation method to detect the differences in response to DC38 stimulation. The results of this study found that PBMCs from MM patients failed to proliferate and secrete IFN*γ* induced by CD38 ligation while retaining their response to TCR/CD3. It was concluded from their findings that CD38 may be functionally involved in the progression of MM via secretion of high levels of IL-6 that protects neoplastic cells from apoptosis.

In the paper entitled “*Differential influence of inositol hexaphosphate on the expression of genes encoding TGF-β isoforms and their receptors in intestinal epithelial cells stimulated with proinflammatory agents*,” M. Kapral et al. studied the effect of inositol hexaphosphate (IP6), a naturally found phytochemical, on lipopolysaccharide- (LPS-) induced TGF-*β* and TGF-*β* receptor expression in intestinal cells. Using qRT-PCR, they found that IP6 inhibited TGF-*β*1, whereas IP6 induced LPS- or IL-1*β*-induced TGF-*β*2 and -*β*3 expression in Caco2 cells. Based on these novel findings, M. Kapral et al. concluded that IP6 elicits immunoregulatory and chemopreventive activity by differentially modulating the expression of TGF-*β*s and their receptor genes.

The paper entitled “*Enhanced inflammatory activity of endometriotic lesions from the rectovaginal septum*” by D. Bertschi et al. is a clinical study which was aimed at understanding the role of chemokines and cytokines in causing inflammation in ectopic and eutopic lesions from the rectovaginal septum. Their study found that the gene expression of chemokines ENA-78 and RANTES and the cytokines IL-6 and TNF-*α* was higher in endometriotic lesions compared to a nonlesion ectopic tissue. The results of this study should provide the impetus for further clinical and/or experimental studies designed to validate these findings and to test whether the inhibition of these cytokines and/or chemokines may be beneficial to the reduction of inflammation in the endometriotic lesions of the rectovaginal septum.

In the paper entitled “*The role of T cell immunoglobulin mucin domains 1 and 4 in a herpes simplex virus-induced Behçet's Disease mouse model*,” J. A. Shim et al. provided novel understanding of the regulatory role of T cell immunoglobulin-1 (TIM-1) and TIM-4 in a mouse model of Behçet's disease. The results of this study showed that siRNA targeting TIM-1 attenuated the symptoms typical of Behçet's disease while also decreasing the severity of the disease. In a parallel study J. A. Shim et al. also found that knockdown of TIM-4 also elicited a beneficial effect. These findings underlined the importance of TIM-1 or TIM-4 as potential therapeutic targets for intervention in Behçet's Disease.

In the paper entitled “*IRF5 is a specific marker of inflammatory macrophages in vivo*,” M. Weiss et al. validated the transcription factor, IRF5, to be a biomarker specific for inflammatory macrophage subset, M1. The study provided evidence that IRF5 regulates the expression of proinflammatory genes such as IL-12*β* and IL-23*α* whilst repressing anti-inflammatory genes such as IL-10. Mouse bone marrow derived macrophage (BMDM) that differentiates into macrophages under granulocyte-macrophage colony-stimulating factor (GM-CSF) stimulation produced high levels of IRF5 mRNA and IRF5 protein while also displaying MHC II expression (a marker of the M1 type), but these cells lacked M2-marker CD206 expression. These findings were further validated in an antigen-induced arthritis model which emphasized the relevance of the* in vitro* studies to physiological conditions. The results of this study established the species-invariant role of IRF5 in controlling the inflammatory phenotype which in the future may have clinical application.

In the study paper “*Modulation of conjunctival goblet cell function by inflammatory cytokines*,” L. Contreras-Ruiz et al. explored a possible role for proinflammatory cytokines in regulating inflammation at ocular surfaces that are associated with mucin secreting goblet cells. In Sjögren's syndrome, primary cultures of mouse goblet cells from conjunctival tissue were developed to determine their responsiveness to cytokines. They found that TNF-*α* and IFN-*γ* induced goblet cell apoptosis as well as inhibiting mucin secretion in response to cholinergic stimulation. Surprisingly, IL-6 enhanced the secretion of mucin, whereas IL-13 and IL-17 had no modulating effect on mucin secretion. These findings identified key proinflammatory cytokines that directly disrupt conjunctival goblet cell functions and contribute to damage at the ocular surface. The further testing of these changes is likely to facilitate our understanding of the underlying mechanism responsible for ocular damage in Sjögren's syndrome.

The paper entitled “*Induction of tumor necrosis factor release from subtypes of T cells by agonists of proteinase activated receptors*” evaluated the role of protein activated receptors (PARs) in mediating TNF-*α* secretion from highly purified T cells obtained from human peripheral blood. The results of this study showed that PAR-1 antagonist, but not PAR-2 antagonist, completely inhibited trypsin- or thrombin-induced TNF-*α* secretion. This analysis study also showed that ERK1/2 and PI3K/Akt pathways were involved in trypsin- or thrombin-induced TNF-*α* release from T cells. Importantly, TNF-*α* section was observed only in CD4^+^, IL-4^+^, or CD25^+^ T cells, but not in IFN^+^ or IL-17^+^ T cells, which underscores the role that PAR-1 plays in the induction of TNF-*α* release from IL-4^+^ and CD25^+^ T cells. Thus, regulating PAR-1-mediated TNF-*α* release may be beneficial in limiting its role in immunological responses and chronic inflammation.

The paper entitled “*The mechanism of sevoflurane preconditioning-induced protections against small intestinal ischemia reperfusion injury is independent of mast cell in rats*” by X. Gan et al. focused on investigating the efficacy of a novel inhaled anesthetic, sevoflurane, in ischemic reperfusion injury (IR) in rats induced by artery occlusion method. The findings of this study suggested that preconditioning with sevoflurane inhibited IR injury primarily by inhibiting the synthesis of p47^phox^ and gp91^phox^, ICAM-1, malondialdehyde, and IL-6. Moreover, mast cells were not involved in this attenuation process.

In the paper entitled “*Progression of luminal breast tumors is promoted by Ménage à Trois between the inflammatory cytokine TNFα and the hormonal and growth-supporting arms of the tumor microenvironment*,” P. Weitzenfeld et al. showed that TNF-*α* may have strong tumor-promoting actions in luminal breast cancer cells when combined with estrogen and EGF, but not when TNF-*α* acts alone. Interestingly, the presence of TNF-*α* along with the other two growth promoting agents was sufficient to convert nonmetastatic tumor cells into a highly aggressive and metastatic phenotype. This novel finding highlights the strong prometastatic ability of TNF-*α* which may warrant testing of the clinically approved inhibitors of TNF-*α* employed for the treatment of rheumatoid arthritis and Crohn's disease to breast cancer.

The paper which is entitled “*Haplotype analysis of interleukin-8 gene polymorphisms in chronic and aggressive periodontitis*” is a clinical study. P. B. Linhartova et al. examined the role of IL-8 gene polymorphisms in chronic and aggressive periodontitis and how these polymorphisms may influence specific pathogen-mediated connective tissue loss and alveolar bone destruction. Thus, an evaluation of the genomic DNA isolated from chronic periodontitis and aggressive periodontitis, and control human subjects identified the rs4073, rs2227307, rs2227306, and rs2227532 gene polymorphisms and showed that the A(−251)/T(+396)/T(+781) and T(−251)/G(+396)/C(+781) haplotypes were significantly less frequent in patients with chronic periodontitis. Thus, the results of this study provide evidence that some of the IL-8 haplotypes could be protective against chronic periodontitis in the affected population.

Although immunotherapies are one of the most promising treatment strategies for cancer, systemic tumor-targeted delivery of these agents has proven to be the biggest obstacle to their clinical success. In the research article entitled “*Intricacies for posttranslational tumor-targeted cytokine gene therapy,*” J. Cutrera et al., using multiple syngeneic murine tumor models, examined the potential impact of the location of the targeting peptide, choice of targeting peptide, tumor histotype, and cytokine utilization on clinical efficacy. Using IL-12 gene therapy as a model system, the authors found that, within the same tumor model, the location of targeting peptide was very critical in achieving a good clinical response.

In the paper entitled “*A possible role of CD8* ^*+*^ *T lymphocytes in the cell-mediated pathogenesis of Pemphigus vulgaris*,” F. Giurdanella et al. examined the role of CD8^+^ T cells in CD8 deficient mice by evaluating the development of acantholysis with passive transfer of pemphigus vulgaris (PV) autoantibodies. The results showed a lower incidence of PV in the CD8 deficient mice compared to their wild-type counterpart. These findings also pointed to the possible role of Fas/FasL death receptor complex and ligand duo in mediating CD8^+^ T cell immune response.

Bronchopulmonary dysplasia (BPD) is a chronic lung disease in neonates primarily caused by inflammation and epithelial cell death from mechanical ventilation and excess oxygen exposure otherwise known as hyperoxia. In the study entitled “*Hyperoxia exacerbates postnatal inflammation-induced lung injury in neonatal BRP-39 null mutant mice promoting the M1 macrophage phenotype*,” M. A. Syed and V. Bhandari studied O_2_-induced macrophage polarization and the anti-inflammatory role of breast regression protein-39 (BRP-39) as causative in neonatal lung injury. The results of their study showed that hyperoxia enhanced LPS-induced M1 polarization while inhibiting the IL-4-induced M2 phenotype. The absence of BRP-39 further enhanced LPS-mediated M1 phenotype macrophage polarization. Furthermore, BRP-39^−/−^ mice also showed a higher sensitivity towards hyperoxia-induced lung injury and higher mortality compared to wild-type mice. Thus, the results of this study highlighted the protective role of BRP-39 in reducing neonatal lung injury by deactivating M1 macrophage-induced inflammatory responses.

In the study entitled “*Chemokines and neurodegeneration in the early stage of experimental ischemic stroke*,” P. Wolinski and A. Glabinski analyzed the expression of several inflammatory biomarkers and correlated these biomarkers with the development of neurodegenerative symptoms in the early phase of experimental stroke. Using endothelin-1- (ET-1-) induced ischemic stroke model, they found that the early phase of experimentally induced stroke was characterized by migration of inflammatory lymphocytes and macrophages to the brain. However, the study did not find any conclusive evidence to correlate this migration with neurodegeneration. These findings suggest that chemokines may be a potential therapeutic target for regulating inflammatory cell accumulation in the early stage of experimentally induced stroke which might minimize ischemic neurodegeneration.

In the study entitled “*Cytokines and chemokines as regulators of skeletal muscle inflammation: presenting the case of Duchenne muscular dystrophy*,” B. D. Paepe and J. L. D. Bleecker reviewed the growing accumulation of evidence for the role of cytokines and chemokines in the pathophysiology in a case of Duchenne muscular dystrophy. For obvious reasons, these findings in this individual case require further validation in animal models of Duchenne muscular dystrophy to justify for future therapeutic development.

The benefits of testosterone replacement therapy (TRT) are still debated due to significant untoward changes that are triggered in the body by TRT including changes in body composition and lipid metabolism, along with decreased high density lipoprotein (HDL), adiponectin, and osteoprotegerin levels. In the study entitled “*Strength training and testosterone treatment have opposing effects on migration inhibitor factor levels in ageing men*,” D. Glintborg et al. studied the clinical effect of TRT gels on strength training (ST) in aged men and the possible role that chemokines play in the process. The results of a double-blinded, placebo-controlled study of six-month TRT therapy showed a decrease in MIF levels in the ST placebo group, compared to increased MIF levels with TRT therapy. Thus, a consistent increase in MIF levels during TRT therapy suggests its possible association with increased inflammatory activity.

In the paper entitled “*Palmitic acid induces production of proinflammatory cytokines interleukin-6, interleukin-1β, and tumor necrosis factor-α via a NF-κB-dependent mechanism in HaCaT keratinocytes*,” B. Zhou et al. studied the effect of palmitic acid on keratinocytes. This study found that palmitic acid treatment induced production of IL-6, TNF-*α*, and IL-1*β* via activation of the NF-*κ*B pathway in human keratinocytes. They also found that palmitic acid upregulated peroxisome proliferator-activated receptor- (PPAR-) *α* activation and the phosphorylation of signal transducers and activators of transcription-3 protein by keratinocytes* in vitro*. These findings provided evidence that blockade of IL-6 via NF-*κ*B pathway was a very effective in regulating palmitic acid-induced inflammation and hyperproliferation in keratinocytes.

We sincerely hope that the present special issue may provide useful information to help understand the mechanisms of inflammation mediated by cytokines or chemokines and potential new therapeutic targets. We hope that the reader will find some novel input for future research.

*Salahuddin Ahmed* *Salahuddin Ahmed* *Charles J. Malemud* *Charles J. Malemud* *Alisa E. Koch* *Alisa E. Koch* *Mohammad Athar* *Mohammad Athar* *Dennis D. Taub* *Dennis D. Taub*

